# Clinical and Economic Impact of Rapid Blood Pathogen Identification Via Verigene

**DOI:** 10.7759/cureus.30366

**Published:** 2022-10-16

**Authors:** Hoa Ngo, Uche J Mbadugha, Frances Cepeda, Salim Surani, George Udeani

**Affiliations:** 1 Pharmacy, AdventHealth Central Texas, Killeen, USA; 2 Pharmacy, Corpus Christi Medical Center, Corpus Christi, USA; 3 Microbiology, Corpus Christi Medical Center, Corpus Christi, USA; 4 Anesthesiology, Mayo Clinic, Rochester, USA; 5 Medicine, Texas A&M University, College Station, USA; 6 Medicine, University of North Texas, Dallas, USA; 7 Internal Medicine, Pulmonary Associates, Corpus Christi, USA; 8 Clinical Medicine, University of Houston, Houston, USA; 9 Pharmacy, Texas A&M Health Science Center, Kingsville, USA

**Keywords:** bloodstream infections, antimicrobial stewardship, verigene, gram negative bactermia, blood culture, infectious disease, bacteremia

## Abstract

Introduction: Bloodstream infections (BSIs) are associated with increased morbidity and mortality if not treated appropriately. Rapid identification of microorganisms will allow clinicians the opportunity to modify initial broad-spectrum antibiotic therapy and improve patient outcomes in bacteremia. We aim to evaluate the impact of the Verigene Gram-positive blood culture (BC-GP) technology on time to modification of antibiotic therapy by clinicians.

Methods: This was a retrospective research study conducted at Corpus Christi Medical Center. Verigene BC-GP technology was employed to rapidly identify microorganisms in patients with suspected Gram-positive bacteremia. Empiric antibiotic therapy was modified via de-escalation or escalation when culture results became available. The primary outcome for this study was the mean time to modification of antibiotic therapy after Verigene BC-GP results became available. Data analysis was conducted from data collected between January 2015 and August 2017 to assess the clinical and pharmacoeconomic impact of BC-GP.

Results: Data were collected on 159 patients, with 123 of 159 (77%) meeting the inclusion criteria. The mean age was 66 ± 14.9 years, with 53/123 (43%) females and 70/123 (57%) males. Positive cultures identified were as follows: *Streptococcus* species (34), *Staphylococcus* species (72), 31/72 (43%) were MRSA, and *Enterococcus* species (19), 4/19 (21%) were Vancomycin-resistant *Enterococcus* (VRE). Antibiotic therapies in 31 of 123 patients (25%) were escalated, and 29 of 123 (24%) were de-escalated. Therapy was determined to be appropriate based on culture results in 63 of 123 (51%) patients, and thus therapy was not modified in this group. The mean time to escalate therapy was 6.2 ± 6 h and 9.2 ± 12.1 h to de-escalate. The average time for modification of antibiotic therapy was 7.6 ± 9.5 h. The conventional approach would take approximately 24-72 h for pathogen identification. Data on cost savings per intervention is estimated to be approximately $4000 per intervention. Based on this model, we estimate approximately $240,000 in cost savings from the 60 cases where interventions occurred.

Conclusion: There is a significant time advantage to pathogen identification, therapy modification as well as a pharmacoeconomic benefit associated with the Verigene GC-GP system as compared to the conventional approach, which translates to positive patient outcomes.

## Introduction

Bloodstream infections (BSIs) are associated with increased morbidity and mortality if not treated appropriately [1-2]. Rapid identification of the offending microorganism has been shown to shorten the length of hospital stay, reduce healthcare costs, and lower mortality rates [3-6]. Empiric broad-spectrum antibiotic therapy is initiated for BSIs to impair or eliminate potential bacteria that could be the culprit of the infection. Initial broad-spectrum antibiotics are warranted, but the need to de-escalate the antibiotics promptly is critical as antibiotic use is associated with various adverse effects, antibiotic resistance, and drug toxicity [7]. According to the United States Centers for Disease Control (CDC) and Prevention, it is estimated in 2013 that there are over two million illnesses associated with antibiotic resistance each year that results in approximately 23,000 deaths [8]. Calculating the medical cost of antimicrobial resistance has been challenging and varies amongst studies but can be as high as $20 billion annually in the United States [8]. A previous study examining six multi-drug resistant (MDR) infections resulted in approximately $1.9 billion in medical costs amongst older adult patients in the United States in 2017 [9]. It was calculated that more than 400,000 inpatient days occurred and 11,852 deaths resulted due to MDR infections in 2017 [9]. The use of any antibiotics also contributes to the cause of *Clostridium difficile* infections where the CDC estimates approximately 250,000 *C. difficile* infections each year which is associated with antibiotic use which result in approximately 14,000 deaths [8]. *C. difficile* infection is a complication that can be prevented if healthcare professionals can de-escalate antibiotics as soon as lab culture results are available and are clinically appropriate. The standard of practice for diagnosing BSIs is through the use of blood cultures. Positive blood cultures are then Gram-stained to determine if the bacteria present is Gram-positive or Gram-negative. Additional growth in vitro (transferring the sample onto agar plates and then incubated) to allow for the identification of the specific microorganism is then required. This process of identifying the microorganism of interest could take anywhere between 24 and 72 h depending on how quickly the microorganisms grow, delaying the opportunity for clinicians to de-escalate or broaden initial empiric antibiotic therapy if necessary [10].

With the rise in antibiotic resistance and the necessity to improve antimicrobial stewardship in the clinical setting, various new technologies have been created to allow the rapid identification of microorganisms and their resistance markers. Previous studies have examined various technologies for the rapid identification of microorganisms in positive cultures and their relationship to hospital length of stay and economic benefits. Peptide nucleic acid (PNA) fluorescent in situ hybridization (FISH) stains is an example of a technology used to rapidly identify pathogens in positive blood cultures [11]. PNA-FISH identifies specific pathogens by targeting specific rRNA that is produced in growing bacteria and yeast. NA-FISH identifies various pathogens including *Staphylococcus aureus, Coagulase-negative staphylococci* (CoNS)*, Enterococcus faecalis, Enterococcus* species*, Escherichia coli, Klebsiella pneumoniae, Pseudomonas aeruginosa,* and *Candida *species [11]. Despite the technology’s ability to identify various microorganisms, PNA-FISH did not have the ability to identify resistance markers that some pathogens may have. Results are available within approximately 90 min. A previous retrospective study was done examining the use of PNA-FISH at a 650-bed academic medical center to look at the median hospital length of stay and economic benefit of the technology [12]. The study determined a decrease in median hospital length of stay by two days and cost savings of approximately $4005 per patient [12].

The Verigene Gram-positive blood culture (BC-GP) test is a multiplex nucleic assay that identifies microorganisms and resistance markers from positive blood cultures [13]. Microorganisms are identified through the presence of specific nucleic acid sequences. The BC-GP assay detects various Gram-positive bacterial pathogens that include *Staphylococcus species, Staphylococcus aureus, Staphylococcus epidermidis, Staphylococcus lugdudensis, Streptococcus* species*, Streptococcus pyogenes, Streptococcus agalactiae, Streptococcus anginosus* group*, Streptococcus pneumoniae, E. faecalis, Enterococcus faecium,* and *Listeria* species. In addition, BC-GP assay also detects the presence of three resistance markers, mecA, vanA, and vanB [13]. When *S. aureus* bacteria carry the mecA gene, the bacteria become MRSA (methicillin-resistant *S. aureus*). The mecA gene allows for the bacteria to encode for the PBP2a (penicillin-binding protein 2a), which has a low affinity for all beta-lactam antibiotics including penicillins, cephalosporins (except the fifth generation), and carbapenems [14]. The vanA and vanB genes are most commonly found in vancomycin-resistant *Staphylococcus aureus* and vancomycin-resistant *Enterococcus*. Vancomycin normally binds to the D-alanine-D-alanine (target site) terminus of bacterial peptidoglycan cell wall. When vanA and vanB genes are present, it alters the terminus into D-alanine-D-lactate, preventing vancomycin from being able to bind to its target site [14]. The BC-GP assay provides results within approximately 2.5 h, which is significantly less time compared to the standard of practice (24-72 h). Previous studies have demonstrated a 92%-95% overall agreement rate for Gram-positive microorganism identification between the BC-GP assay and conventional methods [15-16].

The aim of this study is to evaluate the impact of Verigene BC-GP on time to modification of antibiotic therapy by clinicians.

## Materials and methods

**Figure 1:**
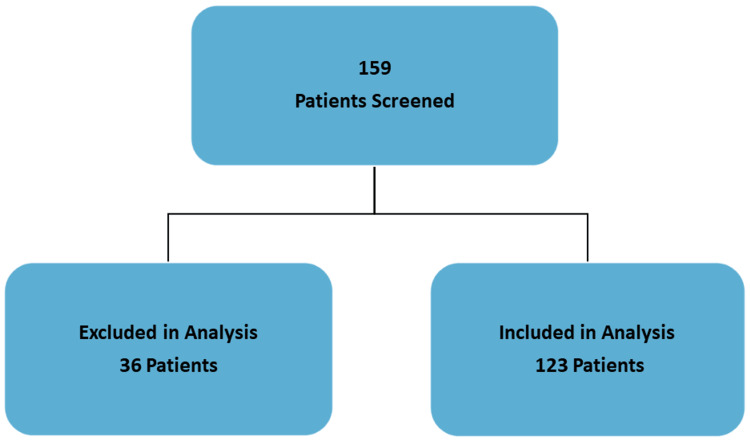
Number of patients who were included and excluded from data analysis.

This was a single-center, retrospective study analyzing the utilization of the Verigene BC-GP technology to help guide antibiotic therapy. The study was conducted at Corpus Christi Medical Center (CCMC) after approval from the Institutional Review Board of CCMC. The primary outcome of the study was to determine the average time it took physicians to modify antibiotic therapy after BC-GP assay results were available. In addition, the secondary outcome will examine the pharmacoeconomic benefits of using the BC-GP technology. Patients were identified by utilizing the microbiology laboratory’s data of patients who had their blood cultures tested with the BC-GP assay. Data were collected and analyzed for January 2015 through August 2017. Patients were included in the study if they were 18 years or older and had positive blood cultures for *Staphylococcus* species, *S. aureus*, *S. epidermidis*, *S. lugdudensis*, *Streptococcus* species, *S. pyogenes*, *S. agalactiae*, *S. anginosus* group, *S. pneumoniae*, *E. faecalis*, *E. faecium*, and *Listeria* species. In addition, patients were excluded if they presented to the emergency department but were not admitted, expired prior to the availability of BC-GP assay results, concomitant Gram-negative infections in the bloodstream, or Gram-positive BSIs not listed in the inclusion criteria (Figure 1). The most common reason for exclusion was emergency room patients who were not admitted.

At CCMC, the BC-GP assay was only utilized if the Gram stain of the blood sample showed Gram-positive microorganisms. The microbiology laboratory only tested blood cultures using the BC-GP assay Monday through Friday between 0600 and 1300 when there was microbiology staff available to read and report the results to the healthcare team. In addition to the BC-GP assay results being reported on the patient’s electronic medical record, the microbiology lab would also attempt to contact the physician to report the results. When the microbiology lab was unable to contact the physician, the patient’s nurse was contacted to follow up with the physician with the results. In 32/123 (26%) cases, the clinical pharmacists were also contacted with the results. Clinical data were collected from the patient’s electronic medical record. The time at which blood cultures were drawn was documented, along with the time when the BC-GP assay results were available to the medical team. Antibiotics that the patient was receiving up until the BC-GP assay results, excluding antibiotics that were given but discontinued prior to results, were also noted. 

Data for this study were analyzed using descriptive statistics for patient characteristics and mean and standard deviation for continuous data that was collected. The time of modification of therapy was determined from the time that the medical team was contacted with the results to the time that the therapy was either de-escalated or escalated. This data was analyzed as a whole to determine the average time that antibiotics were modified and then further broken down to determine the average time to either de-escalate or escalate antibiotics.

## Results

A total of 159 patients with Gram-positive bacteremia were assessed. Due to the exclusion criteria listed above, only 123 patients were included in the data analysis. There was a similar number of males and females included in the study with an average age of 66 years (Table 1).

**Table 1:** Demographic and clinical outcomes.

Patient characteristics (N=123)	
Females	53 (43)
Males	70 (57)
Age (years)	66 ± 14.9
Hospital length of stay (days)	14.6 ± 13.5
Hospital mortality	3 (2)

The majority of blood cultures analyzed grew a *Staphylococcus* species (72) followed by *Streptococcus* species (34) (Table 2). Out of 123 blood cultures, two were found to be polymicrobial with two Gram-positive organisms growing in each blood culture, giving us a total of 125 microorganisms identified. The polymicrobial blood cultures were growing MRSA with Vancomycin-resistant *Enterococcus* (VRE) and MRSA with *E. faecalis*.

**Table 2:** Blood culture results. MRSA, methicillin-resistant *Staphylococcus aureus;* VRE, vancomycin-resistant *Enterococcus*

Microorganisms (N=125)		
Streptococcus	S. pneumoniae	16
	S. agalactiae	14
	S. anginosus	1
	S. pyogenes	2
	Group F *Streptococcus*	1
Staphylococcus	S. aureus	36
	MRSA	31
	S. epidermidis	5
Enterococcus	E. faecalis	15
	VRE	4
Total count		125

The Verigene BC-GP assay does not provide sensitivity, therefore, a change in antibiotic therapy (de-escalation or escalation) is only considered as a modification of therapy due to BC-GP assay results if it occurred prior to the availability of sensitivities. Modification of antibiotic therapy that occurred after sensitivities were available was not considered a modification of antibiotic therapy as a result of the Verigene BC-GP assay.

There were 60 patients who had their antibiotic therapy modified, based on the BC-GP assay results. On average, it took physicians approximately 7.6 ± 9.5 h to modify (de-escalate or escalate) antibiotic therapy (Table 3). Within the subgroup analysis, we determined that 31 patients had their antibiotics escalated with a mean time of 6.2 ± 5.9 h. On the other hand, de-escalation occurred in only 29 patients with a mean time of 9.2 ± 12.1 h. There were 63 patients whose antibiotic therapy was not modified based on BC-GP assay results. The physicians determined that the antibiotic therapies in these 63 patients were appropriate or necessary, and therefore no modifications occurred.

**Table 3:** Mean time to antibiotic modification after blood culture results were available.

Mean time to antibiotic modification (h) (N=123)	
Average time per case (N=60)	7.6 ± 9.5
De-escalation (N=29)	9.2 ± 12.1
Escalation (N=31)	6.2 ± 5.9

Cost savings per intervention is estimated to be approximately $4,000 based on previous studies [15]. With a total of 60 interventions that occurred, we estimate that there were approximately $240,000 in cost savings for the hospital between January 2015 and August 2017 from the utilization of the Verigene BC-GP assay.

## Discussion

Studies have shown a significant difference in time to antimicrobial optimization through the use of the BC-GP assay. A prior study done at Baylor University Medical Center at Dallas analyzed the de-escalation of empiric antibiotic therapy for methicillin-sensitive *S. aureus* (MSSA) and VRE bacteremia, and it was determined that the mean time to the first dose of optimal antibiotic therapy was reduced by 18.9 h when the BC-GP assay was utilized [17]. Another study done at a pediatric hospital demonstrated that the BC-GP assay helped reduce the time to antibiotic optimization by 12.5 h [18]. Our research study at CCMC also resulted in major time differences in antibiotic therapy modification when the BC-GP assay rapid identification technology was used compared to conventional approaches in identifying microorganisms in blood cultures. 

The results of this study showed that on average, antibiotic therapy modifications (de-escalation or escalation) occurred in less than 10 h after BC-GP assay results were reported to the healthcare team. This reduction in time to modification of broad-spectrum antibiotics translated into pharmacoeconomic benefits and improvement in antimicrobial stewardship at CCMC. However, modification of broad-spectrum antibiotics only occurred in less than half of the patient cases analyzed. 

There are some limitations to this research study. Utilization of the BC-GP assay only occurred Monday through Friday between 0600 and 1300 due to the lack of microbiology staff to report the results to the healthcare team. This confounding factor will result in an underestimation of the potential benefits of the BC-GP assay. Furthermore, pharmacists were only contacted in 26% of the cases with results from the BC-GP assay. Initially, pharmacists were not contacted with BC-GP assay results when CCMC first acquired the technology. Pharmacists were only contacted with results in the later portion of the study time period. The lack of pharmacist involvement early on could have potentially affected how quickly antibiotic therapy was optimized.

Another limitation of this study was that there was incomplete data provided by the microbiology lab to identify potential patients who could have been included in the study. Patient medical record numbers associated with specific blood cultures were unavailable for the first quarter of 2017 (January 2017 and March 2017), therefore, we were unable to analyze this data. We also excluded patients who were discharged home from the emergency department but were contacted to return to the hospital to receive IV antibiotics due to their positive blood cultures. It was determined that including these patients would alter the overall benefit of the BC-GP assay due to the delay in the time patients would return to the hospital to receive their intravenous antibiotics. Additionally, the exact time healthcare team members were notified of BC-GP assay results was not always reported. When this time was missing, the time that the microbiology lab called gram stain results to the nurse was used in the data analysis instead. At CCMC, Gram stain results are reported to the patient’s nurse prior to utilizing the BC-GP assay. Using the time that the Gram-stain results were reported to nurses when the actual time the healthcare team was contacted with BC-GP assay results were missing, negatively affects the average time that antibiotic therapies were modified. Modification of therapy was based on the time new antibiotics were scheduled to start and not the time that the new antibiotics were administered.

In addition to these limitations, this study did not have a comparator group to determine statistical significance for the mean time that antibiotics were modified when the conventional method is used vs the BC-GP assay. With the lack of a comparator group, we were unable to directly analyze the impact of the BC-GP assay on patient outcomes vs the conventional method.

There have been studies that showed a reduction in hospital length of stay when the BC-GP assay was used. A previous study analyzing the clinical outcomes of the BC-GP assay to optimize antimicrobial therapy for Enterococcus bacteremia determined that the mean reduction in hospital length of stay was significantly shorter with 21.7 days (P=0.0484) [19]. This study determined that the attributed mortality rates were not significantly different between pre-BC-GP and post-BC-GP groups (2.1% vs 14.2% with P=0.065), but the study was not powered to assess mortality rates [19]. In another study, researchers found that usage of the BC-GP assay resulted in a significant reduction in median hospital length of stay that was 1.5 days (P=0.04) shorter in a general pediatric unit and a median of 5.6 days (P=0.01) shorter when they specifically analyzed *S. aureus* bacteremia [18].

At this time, there are a few studies examining the mortality benefits of the BC-GP assay but with varied outcomes. In a study done by Roshdy and colleagues, there was no difference in mortality between the pre-BC-GP and post-BC-GP groups when they assessed patients with Streptococcus and Enterococcus bacteremia [20]. In another study done by Box and colleagues, it was determined that there was no significant difference in mortality rates (9.1% vs 9.2%, P=0.98) found between the pre-intervention and post-intervention groups with BC-GP [21]. There was also no significant difference found in mortality rates (15% vs 18%, P=0.40) between the pre-BC-GP and post-BC-GP treatment groups in a study done by Neuner and colleagues at Cleveland Clinic [22].

Conversely, there have been studies that demonstrated mortality benefits after the implementation of the BC-GP assay. One study examining 226 patients with *S. aureus* bacteremia using the conventional culture method vs the BC-GP assay showed that there was lower in-hospital mortality (13.2% vs 5.8%, P=0.047) and lower 30-day mortality (17.9% vs 8.3%, P=0.025) [23]. Another study done by Mahrous and colleagues examining the benefits of both Verigene Gram-positive and Gram-negative assays together showed that there was a significant difference in in-hospital mortality (18% vs 10%, P=0.034) in the post-intervention phase [24]. In addition, published literature has clearly shown that there is increased mortality when vancomycin is used to treat MSSA bacteremia vs beta-lactam therapy [25]. The BC-GP assay can also quickly identify the presence of MRSA bacteremia. We can extrapolate this information and confidently say that if MRSA is not identified, empiric vancomycin therapy can be de-escalated to an appropriate beta-lactam antibiotic, which can result in mortality benefits. The differences in findings when assessing for mortality benefits amongst the studies discussed could be due to the patients included and excluded from the studies as well as other outliers.

The BC-GP assay also has some limitations in itself. It was designed to only identify 12 of the most common Gram-positive bacteria that cause bacteremia and is not inclusive of every Gram-positive bacterium such as Micrococcus. Additionally, positive blood culture and a Gram stain confirming the presence of Gram-positive bacteria is required prior to using the assay. 

## Conclusions

The increasing prevalence of MDR bacteria is the result of the misuse of antibiotics. Rapid identification diagnostic technology is becoming increasingly important in all institutions because of the growing problem of antibiotic resistance. There are various rapid microbial identification technologies present at this time including Verigene BC-GP and Gram-negative blood culture (BC-GN) assays, PNA-FISH, BioFire FilmArray systems, and many others that vary in what bacteria they can identify and how quickly. Unfortunately, this technology is not available at all institutions. At CCMC, Verigene BC-GP was an expensive technology incorporated into the hospital’s budget and required a change in the workflow and staffing of the microbiology department. It may be due to these same reasons that such technology is not universal yet at all institutions. The use of rapid identification technology can help improve antimicrobial stewardship, reduce the risk of antibiotic adverse events, and prevent complications associated with antibiotic use, such as *C. difficile* infections. At CCMC, the Verigene GC-GP assay has the potential to help guide physicians, pharmacists, and other healthcare providers in preventing the inappropriate use of broad-spectrum antibiotics to improve patient outcomes and reduce healthcare costs. Due to the lack of studies evaluating the direct impact of BC-GP on various aspects of patient outcomes, more studies are needed to address its potential benefits.
